# Stimulus-dependent dissociation between XB130 and Tks5 scaffold proteins promotes airway epithelial cell migration

**DOI:** 10.18632/oncotarget.13261

**Published:** 2016-11-09

**Authors:** Serisha Moodley, Mathieu Derouet, Xiao Hui Bai, Feng Xu, Andras Kapus, Burton B. Yang, Mingyao Liu

**Affiliations:** ^1^ Institute of Medical Science, Faculty of Medicine, University of Toronto, Toronto, Canada; ^2^ Latner Thoracic Surgery Research Laboratories, Toronto General Research Institute, University Health Network, Toronto, Canada; ^3^ Advanced Optical Microscopy Facility, UHN, Toronto, Canada; ^4^ Keenan Research Centre for Biomedical Science, St. Michael's Hospital, Toronto, Canada; ^5^ Sunnybrook Research Institute, Sunnybrook Health Sciences Centre, Toronto, Canada

**Keywords:** actin filament associate protein 1-like 2, SH3PXD2A, lamellipodia, podosomes, lung repair, Pathology Section

## Abstract

Repair of airway epithelium after injury requires migration of neighboring epithelial cells to injured areas. However, the molecular mechanisms regulating airway epithelial cell migration is not well defined. We have previously shown that XB130, a scaffold protein, is required for airway epithelial repair and regeneration *in vivo*, and interaction between XB130 and another scaffold protein, Tks5, regulates cell proliferation and survival in human bronchial epithelial cells. The objective of the present study was to determine the role of XB130 and Tks5 interaction in airway epithelial cell migration. Interestingly, we found that XB130 only promotes lateral cell migration, whereas, Tks5 promotes cell migration/invasion via proteolysis of extracellular matrix. Upon stimulation with EGF, PKC activator phorbol 12, 13-dibutyrate or a nicotinic acetylcholine receptor ligand, XB130 and Tks5 translocated to the cell membrane in a stimulus-dependent manner. The translocation and distribution of XB130 is similar to lamellipodial marker, WAVE2; whereas Tks5 is similar to podosome marker, N-WASP. Over-expression of XB130 or Tks5 alone enhances cell migration, whereas co-expression of both XB130 and Tks5 inhibits cell migration processes and signaling. Furthermore, XB130 interacts with Rac1 whereas Tks5 interacts with Cdc42 to promote Rho GTPase activity. Our results suggest that dissociation between XB130 and Tks5 may facilitate lateral cell migration via XB130/Rac1, and vertical cell migration via Tks5/Cdc42. These molecular mechanisms will help our understanding of airway epithelial repair and regeneration.

## INTRODUCTION

The airway epithelium is the first line of defense in response to various chemical, physical and inflammatory insults, and depending on the severity of injury, endures surface epithelium permeability, cell death and denudation of the epithelial cell lining [1]. Airway epithelial cell migration is an early event for repair and regeneration of epithelium after injury [1]. Deregulated cell migration contributes to aberrant airway remodeling resulting in the development of lung disorders [2]. Unfortunately, our current understanding of the molecular processes that regulate airway epithelial cell migration is limited.

Complex cellular processes, like cell migration, are mediated by a network of proteins. Scaffold proteins (also known as signal transduction adaptor proteins) are multi-modular proteins that lack enzymatic function but contain a variety of molecular binding domains and motifs to initiate the translocation, assembly and disassembly of macromolecular complexes [3]. Scaffold proteins are critical for coordinating the interactions and transport of proteins, a key role that couples signal transduction events to regulated cell function [3]. Several scaffold proteins have been implicated in the regulation of cell migration, such as the growth factor receptor-bound protein (Grb) family, tyrosine kinase substrate (Tks) family and non-catalytic region of tyrosine kinase adaptor protein (Nck) family [4-6]. XB130 is a scaffold protein that belongs to the actin filament associated protein family and acts as a signal transduction molecule to mediate cell growth, survival and cytoskeleton remodeling [7].

XB130 contains multiple molecular binding domains that promote protein-protein and protein-lipid interactions, such as Src homology 2 (SH2) and Src homology 3 (SH3) binding motifs and pleckstrin homology (PH) domains [8]. XB130 interacts with several proteins, including Src, Lck, RET/PTC and phosphoinositide 3-kinase (PI3K) [8, 9]. Through these interactions, XB130 regulates cell cycle progression and survival by modulating the activity of PI3K/Akt downstream signals, such as p21Cip1/WAF1, p27Kip1, FOXO3a, GSK3β and caspase 8 and 9 [10]. XB130 is also involved in cytoskeleton reorganization, and it translocates to actin-rich lamellipodia at the cell periphery after stimulation with EGF [11]. Moreover, changes in XB130 expression and structure modulate actin dynamics, cell spreading and migration in cancer cells [11]. XB130 is expressed in airway epithelial cells, and XB130 deficiency in mice affects tracheal epithelial differentiation during airway repair [12]. The presence of XB130 in bronchial epithelial cells promotes proliferation of bronchioalveolar stem cells and Club cells during airway repair and regeneration[13]. XB130 deficiency also enhances septic response and lung injury [14]. In human normal bronchiolar epithelial (BEAS-2B) cells XB130 is involved in nicotine derived nitrosamine ketone (NNK)-induced cell migration [15].

We recently showed that XB130 interacts with another scaffold protein, tyrosine kinase substrate with five SH3 domains (Tks5) [16]. Tks5 contains several molecular binding domains, including a phox homology domain for phosphatidylinositol lipid binding and five SH3 domains that facilitate distinct protein-protein interactions [17]. Tks5 is a cytoplasmic protein, but upon stimulation it translocates to the cell membrane, where it predominantly localizes to podosomes/ invadopodia, which are actin-rich, cytoskeletal structures that are involved in cell migration via proteolytic degradation of extracellular matrix [18]. We have demonstrated that Tks5′s fifth SH3 domain binds specifically to the N-terminal polyproline-rich motif of XB130 [16]. XB130 and Tks5 interaction mediates EGF-induced proliferation and survival of BEAS-2B cells [16].

In this study, we hypothesized that XB130 and Tks5 regulate cell migration in normal human bronchial epithelial cells. We utilized different agents that stimulate signaling events and are associated with lung epithelial injury and repair: PKC activator (phorbol 12, 13-dibutyrate, PDBu), growth factor (EGF) and a nicotine derivative (NNK). PKC activation is a key signaling event in lung epithelial injury and inflammation [1]. PDBu has been shown to promote cell migration through the formation of podosomes and secretion of proteases in lung epithelial cells [19]. EGF is important for cell growth and survival but has also been shown to induce rat alveolar epithelial cell migration [20, 21]. NNK is a cigarette smoking toxin that binds to the nicotinic acetylcholine receptor (nAchR) and has been shown to induce lung injury and cell migration in lung epithelial cells [15, 22]. Both EGFR and nAchR activation are known to stimulate the Src/PI3K and PKC pathways but also interact with and regulate distinct downstream effectors for the modification of cell growth, survival and migration [23, 24]. In the present study, we characterize distinct signaling and functional roles of XB130 and Tks5 scaffold proteins in EGFR and nAchR-induced lung epithelial cell motility.

## RESULTS

### Tks5, but not XB130, is involved in podosome formation and ECM degradation

**Figure 1:**
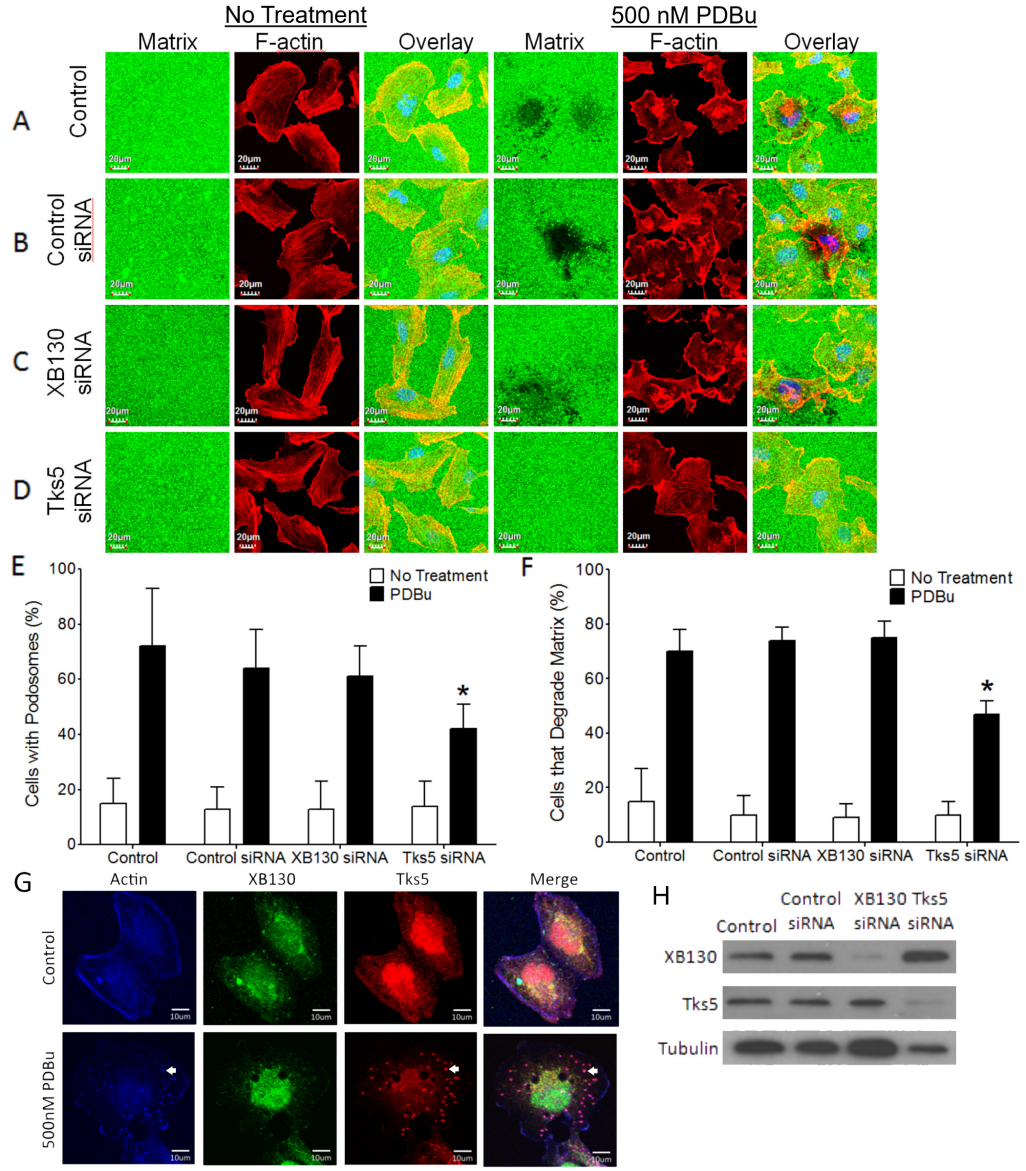
XB130, unlike Tks5, does not play a role in podosome formation and ECM degradation **A.**-**D.** Fluorescent gelatin zymography assay of BEAS-2B cells transfected with control, XB130 or Tks5 siRNA and treated with 500 nM phorbol 12, 13-dibutyrate (PDBu) for 8 h. Cells were stained with rhodamine phalloidin for F-actin (red). Cells were unable to degrade gelatin (green) under basal conditions. PDBu stimulation induced podosome formation (F-actin rich puncta) and gelatin degradation (dark areas). After PDBu treatment, only Tks5 siRNA transfected cells were unable to form podosomes and degrade the gelatin. XB130 siRNA transfected cells behaved like control and control siRNA transfected cells with no effect on the formation or degradation of the gelatin matrix. **E.** Tks5 downregulation significantly reduces the percentage of cells displaying podosomes, after PDBu treatment, whereas XB130 downregulation did not significantly alter percentage of cells with podosomes. **F.** Tks5 downregulation significantly reduces the percentage of cells that are able to degrade gelatin, after PDBu treatment. In contrast to Tks5, XB130 downregulation did not alter the percentage of cells able to degrade gelatin. Data is summarized from three independent experiments and presented as mean ± SD. *p < 0.01 compared with controls (non-transfected BEAS-2B cells and non-targeting siRNA-transfected cells). **G.** Co-immunofluorescence staining of XB130 (green), actin (blue) and Tks5 (red). BEAS2B cells were treated with or without 500 nM PDBu. No treatment control shows normal stress fibers. PDBu treatment shows formation of podosomes (white arrows) as detected by actin and Tks5. XB130 only localizes to the cytoplasm and cell periphery. **H.** Validation of XB130 or Tks5 siRNA transfection using western blot detection of XB130 and Tks5. Tubulin was used as a representative housekeeping protein.

Lung epithelial cell injury due to exposure to environmental insults results in the activation of PKC [1, 25]. The PKC activator, (PDBu), induces cell migration, cytoskeleton remodeling and formation of actin-rich, ventral protrusions of the cell, known as podosomes in BEAS-2B cells [19, 26, 27]. Tks5 has been shown to be involved in cytoskeletal remodeling for the formation of podosomes that secrete proteolytic enzymes for the degradation of the extracellular matrix (ECM) [18]. To determine whether XB130 or Tks5 is involved in podosome formation and function in airway epithelial cells, we performed a fluorescent gelatin degradation assay. BEAS-2B cells were seeded onto coverslips coated with a thin fluorescent gelatin film, which served as a substrate for proteases secreted by migrating cells. Under normal growth conditions, fluorescent gelatin layers appeared evenly coated and cells displayed elongated, parallel stress fibers (Figure 1A & 1B). After stimulation with PDBu, many cells formed ruffled edges at the periphery and punctate actin structures within the cell, indicative of podosomes (Figure 1A & 1B). We observed dark areas in the gelatin film beneath and adjacent to several cells containing podosomes, indicating fluorescent gelatin degradation (Figure 1A & 1B). XB130 and Tks5 but not control siRNA specifically reduced XB130 or Tks5 protein expression (Figure 1H). Tks5 siRNA transfected cells formed fewer podosomes and often failed to degrade the gelatin matrix, in response to PDBu (Figure 1D-1F). In contrast, XB130 siRNA transfected cells displayed very similar morphology and gelatin degradation as compared to control cells (Figure 1C & 1E). Immunofluorescence staining of endogenous XB130 and Tks5 show that both XB130 and Tks5 are normally localized to the cytoplasm and perinuclear region (Figure 1G). However, after PDBu treatment, Tks5 translocates to actin-rich puncta (white arrows), whereas XB130 translocates to actin-associated bands at the cell periphery (Figure 1G).

### XB130 is an essential mediator of lamellipodia formation in airway epithelial cells

**Figure 2:**
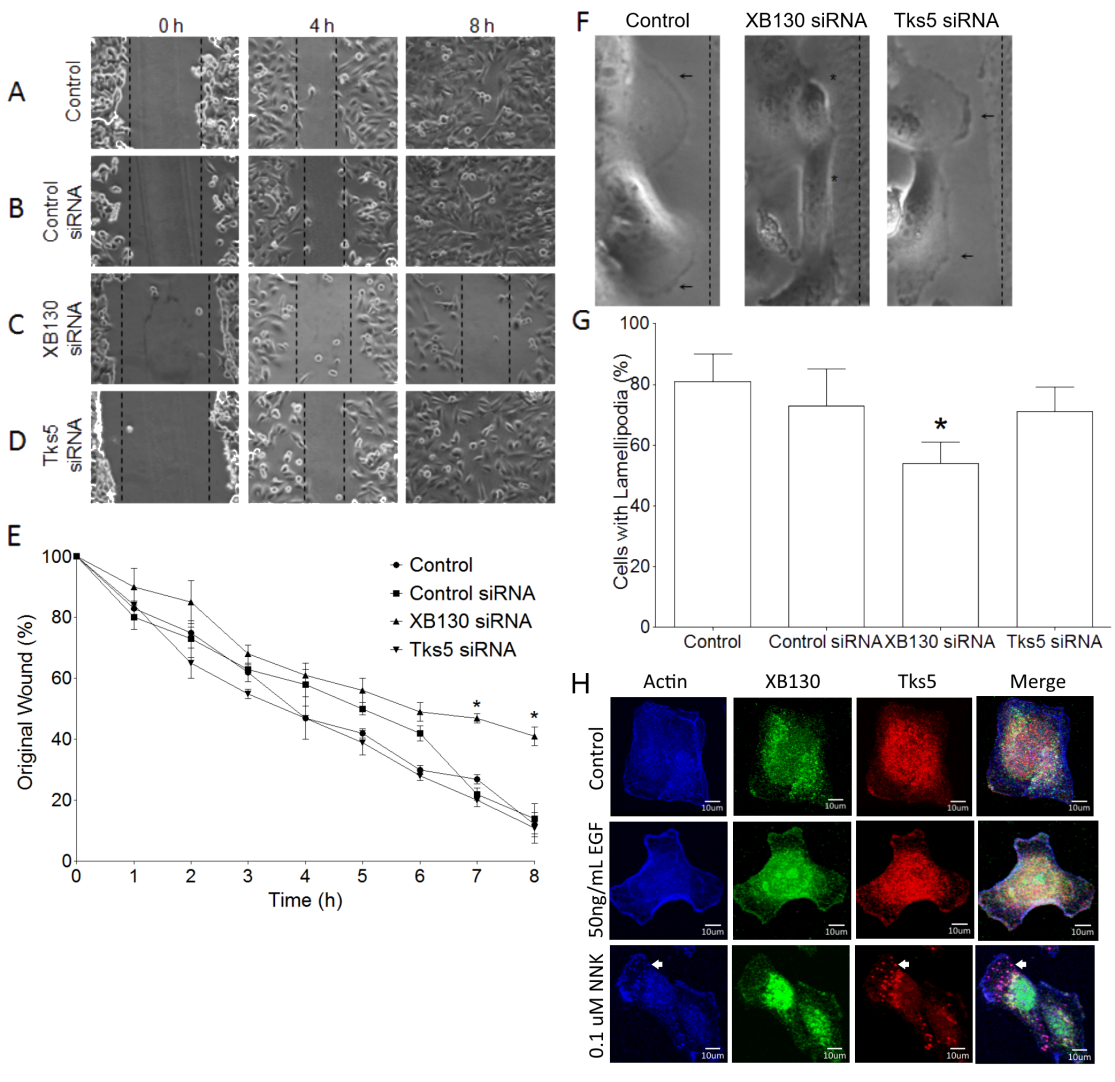
Tks5 is not essential for lamellipodia formation **A.**-**D.** BEAS-2B cells were transfected with control (scrambled), XB130 or Tks5 siRNA and subjected to a wound-healing assay over an 8 h time course. Unlike XB130 down regulation, Tks5 down regulation does not inhibit wound closure. **E.** Percentage of original wound width per hour shows that only XB130 siRNA significantly reduces wound healing at 7 h and 8 h, as compared to control, control siRNA-transfected or Tks5 siRNA-transfected cells. **F.** High magnification phase contrast microscopy at the leading edge of wounds shows that control and Tks5 downregulated cells form dark ruffled edges (arrow), indicative of lamellipodia, whereas XB130 down-regulated cells appear to lack these structures (asterisk). **G.** Quantification of cells with lamellipodia at the leading edge shows that XB130 downregulation significantly reduced the percentage of cells displaying lamellipodia, as observed by phase contrast microscopy. Data is summarized from three independent experiments and presented as mean ± SD. * represents *p* < 0.01 compared with controls (non-transfected BEAS-2B cells and non-targeting siRNA-transfected cells). **H.** Co-immunofluorescence staining of XB130 (green), actin (blue) and Tks5 (red). BEAS2B cells were treated with or without 50 ng/mL EGF or 0.1 uM NNK. No treatment control shows normal stress fibers. EGF stimulation shows formation of lamellipodia as detected by actin bands at the cell periphery and XB130 staining. NNK stimulation shows the formation of lamellipodia and podosomes (white arrows) as detected by actin and Tks5. XB130 only translocates to lamellipodia after stimulation.

Since XB130 is not critical for podosome formation and ECM degradation, like Tks5, we then compared the roles of XB130 and Tks5 in the promotion of lateral cell migration and the formation of lamellipodia, using a wound healing assay. BEAS-2B cells showed progressive migration across the wounded area with about 50% of the original wound width remaining at 4 h and complete closure of the wound at 8 h (Figure 2A & 2E). Cells transfected with a non-targeting scrambled siRNA (Control siRNA) emulated the non-transfected BEAS-2B cells’ migration pattern (Figure 2B & 2E). Cells transfected with XB130 siRNA showed inhibited cell migration starting from 4 h with significant inhibition observed at 7 h and 8 h; the wound width was approximately 50% of the original width at 8 h (Figure 2C & 2E). Interestingly, siRNA down-regulation of Tks5 had no effect on wound closure, as compared to control (Figure 2D & 2E). Using differential interference contrast microscopy, we observed dark ruffled areas indicative of lamellipodia at the front periphery of migrating control cells and Tks5 siRNA transfected cells, whereas, in the XB130 knockdown cells, lamellipodia appeared to be absent or reduced in many cells (Figure 2F). Quantitative analysis of control cells, control siRNA, XB130 siRNA or Tks5 siRNA transfected cells at the leading wound edge showed that only XB130 siRNA down-regulation significantly reduced lamellipodial formation (Figure 2G). Immunofluorescence staining of endogenous XB130 and Tks5 showed that both proteins are expressed in the cytoplasm under control conditions. After 50 ng/mL EGF stimulation, XB130 and Tks5 colocalize with actin-rich bands at the cell periphery, indicative of lamellipodia (Figure 2H). Interestingly, like the PDBu stimulation, 0.1 uM NNK stimulation shows that endogenous XB130 maintains its translocation to the cell periphery, whereas, Tks5 alone translocates to actin-rich puncta (Figure 2H, white arrows). These results validate that XB130 and Tks5 play distinct roles in airway epithelial cell migration; XB130 is critical for lateral migration, whereas Tks5 is involved in cell migration processes coupled to matrix degradation.

### XB130 & Tks5 differentially translocate to cell membrane in a stimulus-dependent manner

**Figure 3:**
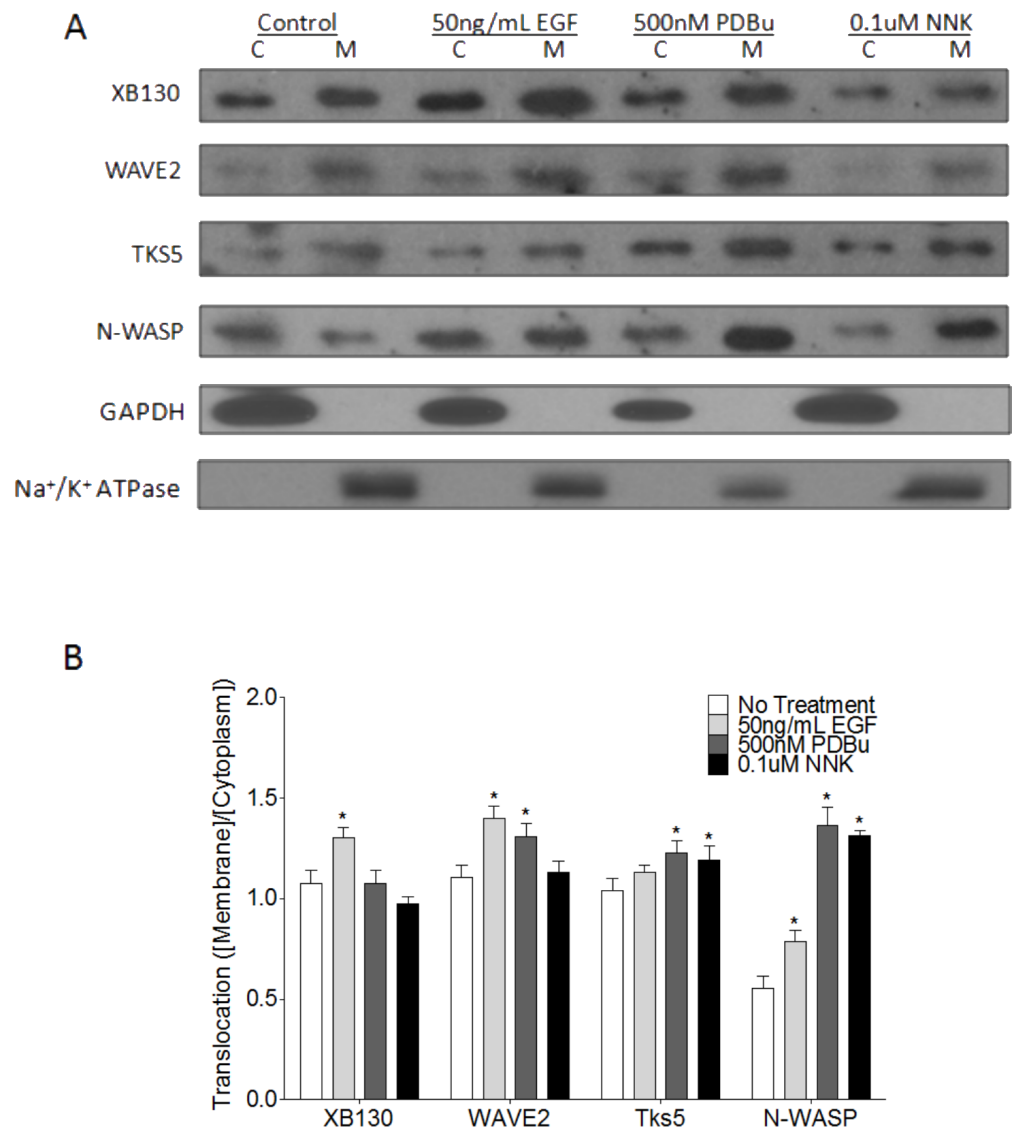
Stimulus-dependent translocation of endogenous XB130 and Tks5 to the cell membrane indicates distinct signaling roles **A.** Immunoblots of cytoplasm (C) and membrane (M) fractionated BEAS-2B cell lysates. Cells were treated with or without 50 ng/mL EGF, 500 nM PDBu or 0.1 μM NNK. XB130 and WAVE2 expression and translocation from the cytoplasm to the cell membrane are more dependent on EGF stimulation, whereas, Tks5 and N-WASP expression and translocation are more dependent on PDBu and NNK stimulation. **B.** Ratio of normalized membrane expression to normalized cytoplasm expression. Expression of Na+/K+ ATPase was used to normalize membrane fractions and expression of GAPDH was used to normalize cytoplasmic fractions. Data is summarized from three independent experiments and presented as mean ± SD. * represents *p* < 0.01 compared to the corresponding no treatment group.

Extracellular stimulation mediates the translocation and protein-protein binding of scaffold proteins, like XB130 and Tks5, for the induction of specific signal transduction pathways and promotion of cell processes. To further confirm that stimulation induces differential expression and localization of XB130 or Tks5 to the cell periphery (Figure 1G & 2H), BEAS-2B cells were stimulated with either 50 ng/mL EGF, 500 nM PDBu, or 0.1 μM NNK, and whole cell lysates were fractionated into cytoplasm and cell membrane for immunoblot detection of XB130 and Tks5. Quantitation of XB130 intensity (normalized to the membrane marker Na+/K+ ATPase and cytoplasmic marker, GAPDH) showed that XB130 translocation to the cell membrane was significantly increased after EGF stimulation, as compared to PDBu and NNK stimulation (Figure 3B). These changes were very similar to the translocation of WAVE2 (Figure 3B), a marker for lamellipodia. In contrast, PDBu and NNK stimulation significantly increased translocation of Tks5 to the cell membrane (Figure 3B), which was similar to translocation of N-WASP, a marker of podosomes (Figure 3B).

### XB130 colocalizes more robustly with lamellipodial marker, WAVE2, than with podosome marker, N-WASP

**Figure 4:**
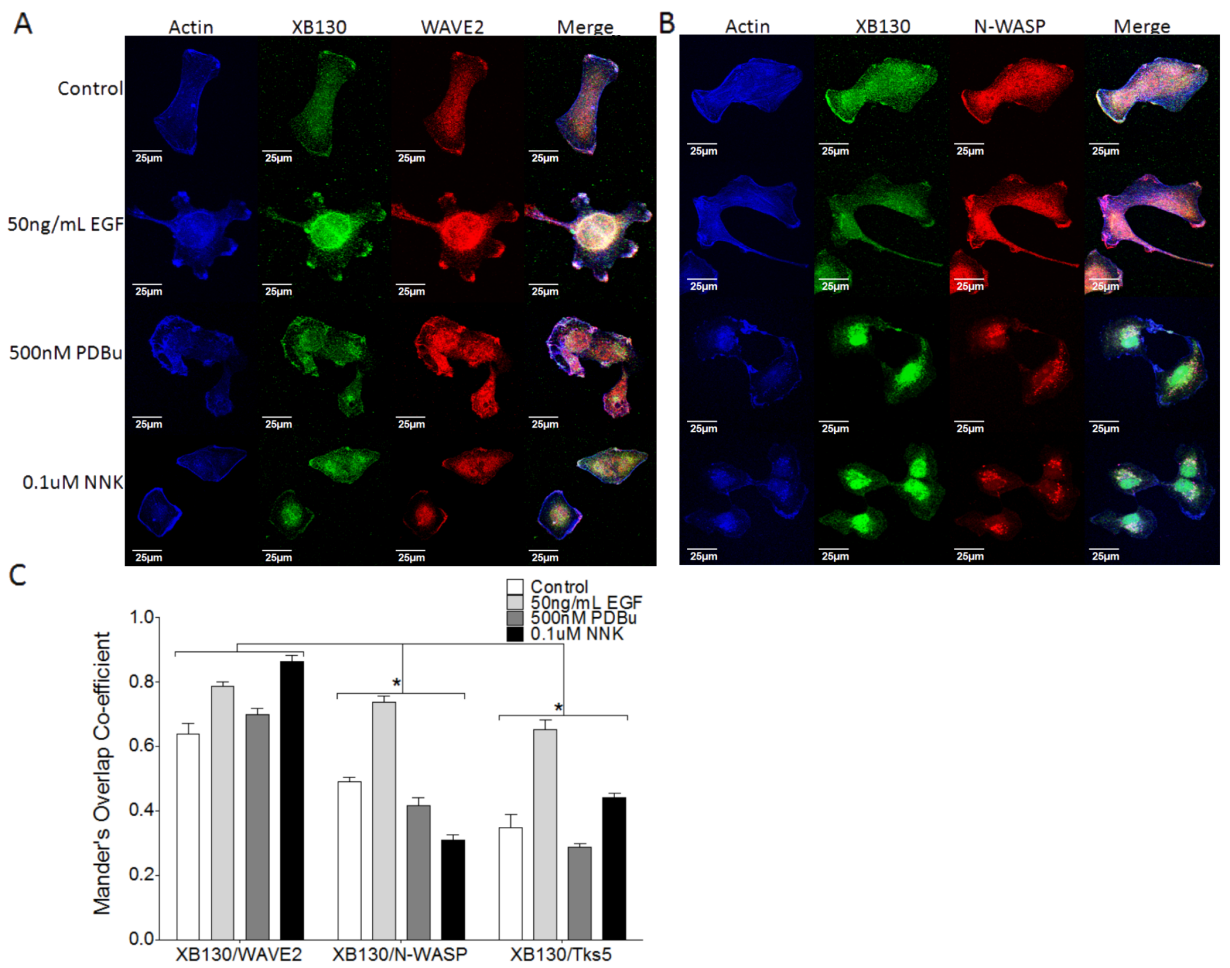
XB130 colocalizes robustly with WAVE2 at lamellipodia but not at podosomes with N-WASP, after stimulation **A.**-**B.** Co-immunofluorescence staining of XB130 (green), actin (blue) and either WAVE2 (A) or N-WASP (B) (red). BEAS2B cells were treated with or without 50 ng/mL EGF, 500 nM PDBu or 0.1 μM NNK. No treatment control shows normal stress fibers. Stimulation with EGF, PDBu and NNK produces actin-rich ruffled areas at the cell membrane, which are indicative of lamellipodia via WAVE2 staining (A). These areas are also enriched with XB130 (A and B). PDBu and NNK induce formation of podosomes (white arrows) which are enriched by N-WASP but not XB130 (B). **D.** Mander's overlap co-efficient (MOC) of the cell periphery displays the relative colocalization of XB130 with WAVE2, Tks5 or N-WASP, where 0 represents no colocalization and 1 represents perfect colocalization. XB130 colocalizes robustly with WAVE2 at the lamellipodia and to a lesser extent with Tks5 and N-WASP, indicating it translocates to and is involved in lamellipodia formation. Data is summarized from 10 different cells per group from 3 different experiments and presented as mean ± SD. * represents *p* < 0.01 for XB130/N-WASP and XB130/Tks5 MOCs compared to XB130/WAVE2 MOCs.

To evaluate the role of XB130 as a lamellipodia marker in contrast to podosomes, we performed immunofluorescence confocal microscopy after stimulation of cells with EGF, PDBu or NNK, and staining for endogenous expression of the lamellipodial marker, WAVE2 or the podosome marker, N-WASP. Under normal growth conditions, endogenous XB130, WAVE2 and N-WASP were mainly localized in the cytoplasm and also at the periphery of the cell (Figure 4A). As expected, WAVE2 was colocalized with actin-rich lamellipodia after stimulation of cells with EGF, PDBu or NNK (Figure 4A). Colocalization between XB130 and WAVE2 at the cell periphery increased, especially after EGF and NNK treatment with a Mander's overlap co-efficient (MOC) at the cell periphery of 0.79 ± 0.025 and 0.86 ± 0.035, respectively, compared with untreated cells with a MOC at the cell periphery of 0.64 ± 0.056 (Figure 4C). After EGF stimulation, XB130 also colocalized with endogenous Tks5 (Figure 2H) and N-WASP (Figure 4B) at the cell periphery, with MOCs of 0.65 ± 0.051 and 0.78 ± 0.021, respectively (Figure 4C). PDBu and, to a lesser extent, NNK, induced formation of actin-rich podosomes, as highlighted by clustered N-WASP (Figure 4B) and Tks5 (Figure 1G) staining. However, XB130 was not observed to colocalize with N-WASP (Figure 4B) nor Tks5 (Figures 1G and 2H) at podosomes. After PDBu and NNK stimulation, colocalization between XB130 and Tks5 or between XB130 and N-WASP was significantly lower than MOCs between XB130 and WAVE2, at the cell periphery (Figure 4C).

### Over-expression of XB130 or Tks5 alone, but not together, enhances lung epithelial cell migration

**Figure 5:**
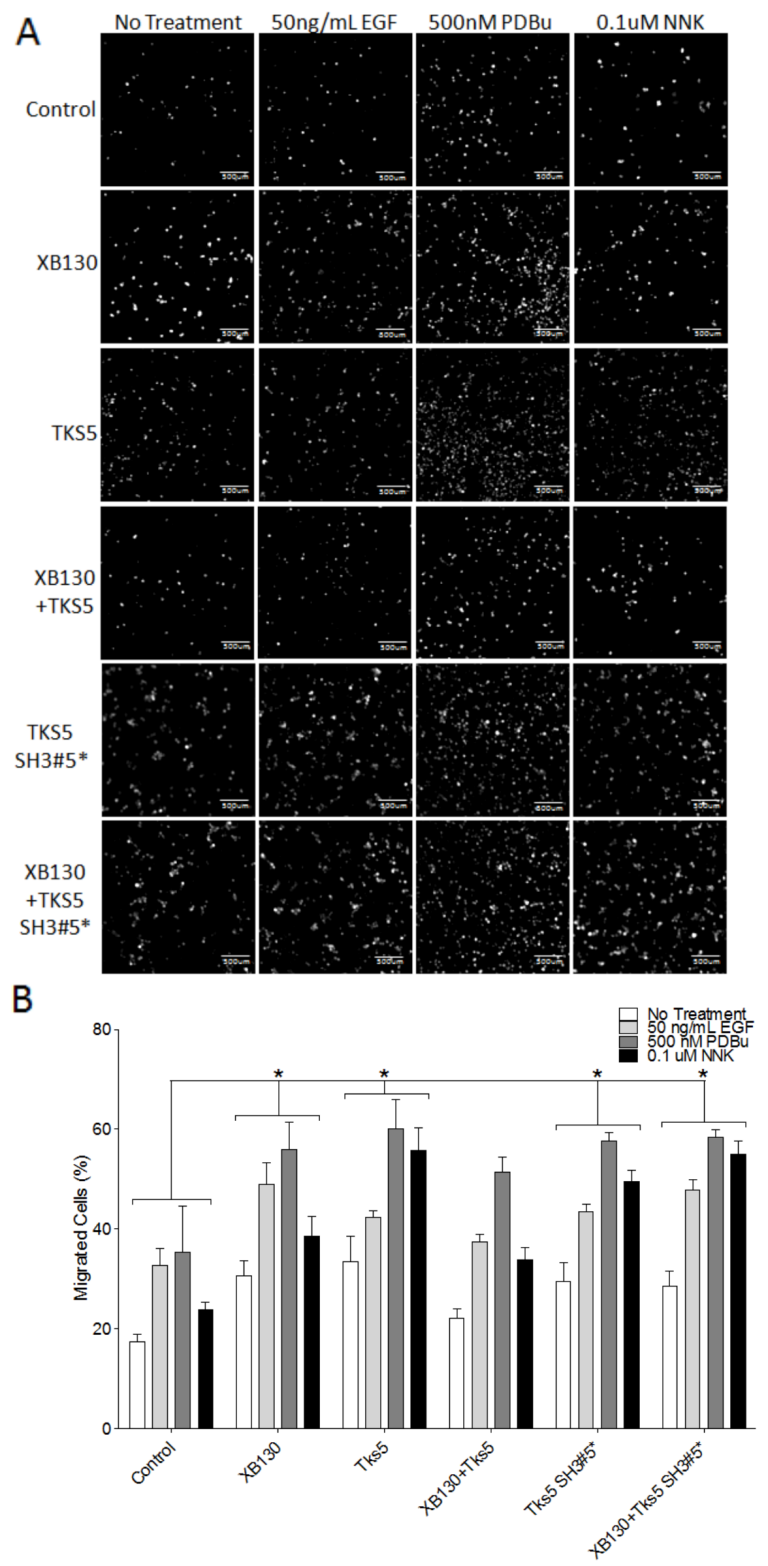
Co-expression of XB130 and Tks5 inhibit the enhanced cell migration observed in cells overexpressing XB130 or Tks5 alone Double chamber transwell cell migration assay using cells transfected with GFP/mCherry-vectors (Control), GFP-XB130/mCherry, GFP/mCherry-Tks5, GFP-XB130/mCherry-Tks5, GFP/mCherry/Myc-Tks5 SH3#5* or GFP-XB130/mCherry/Myc-Tks5 SH3#5*. Cells were stimulated with 50 ng/mL EGF, 500 nM PDBu or 0.1 μM NNK for 1 h. FBS was added to the lower chamber as a chemoattractant. **A.** Images of cells that migrated past the transwell membrane. Scale bars represent 500 μm. **B.** Percentage of migrated cells past the transwell membrane. Over-expression of XB130 or Tks5 alone or Tks5 SH3#5* with or without XB130 significantly increases cell migration, whereas, co-expression of both XB130 and Tks5 has only a moderate increase in cell migration as compared to control cells, after stimulation. Data is summarized from three independent experiments and presented as mean ± SD. * represents *p* < 0.01 of XB130 or Tks5 over-expressing groups versus the control vector-transfected group.

XB130 and Tks5 interact endogenously to mediate cell proliferation, growth and survival, in BEAS-2B cells [16]. To characterize the functional role of the XB130 and Tks5 interaction in cell migration processes, we over-expressed GFP-XB130 and/or mCherry-Tks5 in BEAS-2B cells and evaluated cell migration using double chamber transwell assays. GFP and/or mCherry expressing cells were sorted and collected using flow cytometry. Sorted cells were seeded onto 0.8 μm pore transwells, serum starved and stimulated with 50 ng/mL EGF, 500 nM PDBu or 0.1 μM NNK. Uncoated transwells were incubated in media containing 10% FBS for 8 h to evaluate cell migration. Stimulation with EGF or PDBu increased cell migration in GFP/mCherry vector expressing cells (Control) (Figure 5). Cell migration was significantly enhanced by over-expression of either XB130 or Tks5 alone, whereas co-expression of both XB130 and Tks5 did not show a significant increase in cell migration, as compared to control cells (Figure 5). To test whether co-expression of XB130 and Tks5 inhibited over expression of XB130- or Tks5-induced promotion of cell migration, we expressed Tks5 single amino acid substitution mutant W1108A in the fifth SH3 domain (Tks5 SH3#5*), which we previously demonstrated to block binding between XB130 and Tks5 [16]. Overexpression of the Tks5 SH3#5* mutant enhanced cell migration in a similar manner as Tks5 alone. Furthermore, expression of the Tks5 SH3#5* mutant with XB130 also enhanced cell migration compared to control or XB130 and Tks5 co-expressing cells (Figure 5).

**Figure 6:**
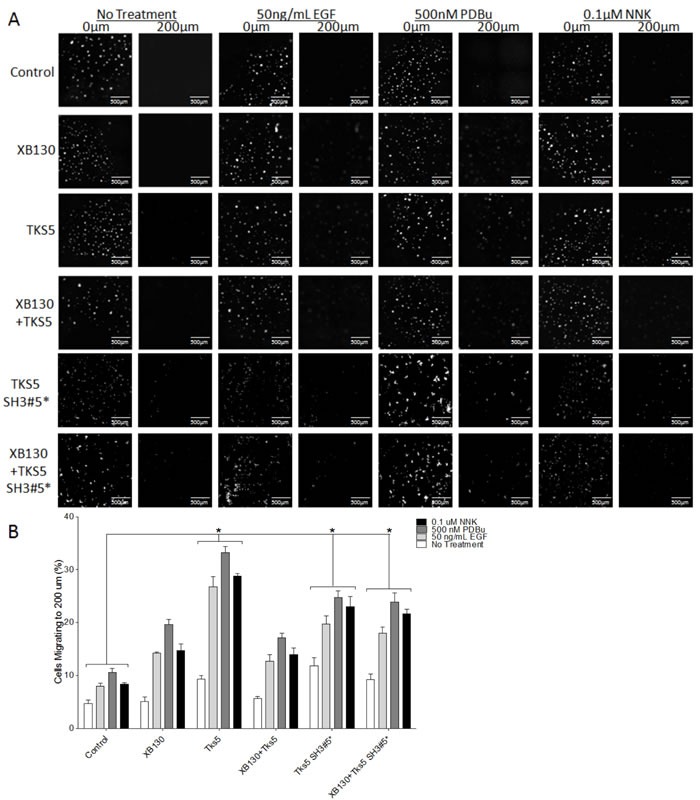
Co-expression of XB130 with Tks5 inhibits the enhanced ECM-dependent cell migration observed in cells over-expressing Tks5 only Matrigel-coated double chamber transwell cell migration assay using cells transfected with GFP/mCherry-vectors (Control), GFP-XB130/mCherry, GFP/mCherry-Tks5, and GFP-XB130/mCherry-Tks5. Cells were stimulated with 50 ng/mL EGF, 500 nM PDBu or 0.1 μM NNK for 1 h. FBS was added to the lower chamber as a chemoattractant. **A.** Images of cells cultured on a thin matrigel coating at 0 μm and migration to 200 μm. Scale bars represent 500 μm. **B.** Percentage of cells migrating to a depth of 200 μm of matrigel. Over-expression of Tks5 alone or Tks5 SH3#5* with or without XB130 significantly increases cell migration into matrigel, whereas, over-expression of XB130 alone does not significantly increase ECM-dependent cell migration, after stimulation, as compared to control cells. Cells co-expressing XB130 with Tks5 show a similar percentage of migrating cells as XB130 over-expression alone. The over-expression of XB130 appears to inhibit Tks5-mediated cell migration, indicating a regulatory effect of XB130 on Tks5. Data is summarized from three independent experiments and presented as mean ± SD. * represents *p* < 0.01 of the Tks5 over-expressing groups versus vector-transfected control group.

We also used a modified matrigel transwell assay to determine the role of XB130 and Tks5 interaction in ECM degradation-dependent cell migration. XB130 over-expression alone did not significantly enhanced ECM degradation-dependent cell migration, while Tks5 or Tks5 SH3#5* over-expressing cells demonstrated robust cell migration through matrigel compared to vector-transfected control cells (Figure 6). Co-expression of XB130 with Tks5 did not increase ECM degradation-dependent cell migration and was significantly reduced as compared to cells over-expressing Tks5 alone (Figure 6). However, co-expression of XB130 with Tks5 SH3#5* mutant led to increased ECM degradation-dependent cell migration compared to control or XB130 and Tks5 co-expressing cells (Figure 6). In both models, overexpression of XB130 and Tks5 together appears to inhibit the enhanced cell migration seen in cells over-expressing either XB130 or Tks5 alone (Figure 5B & 6B).

### XB130 interacts with and activates Rac1 whereas Tks5 mediates Cdc42 activation

**Figure 7:**
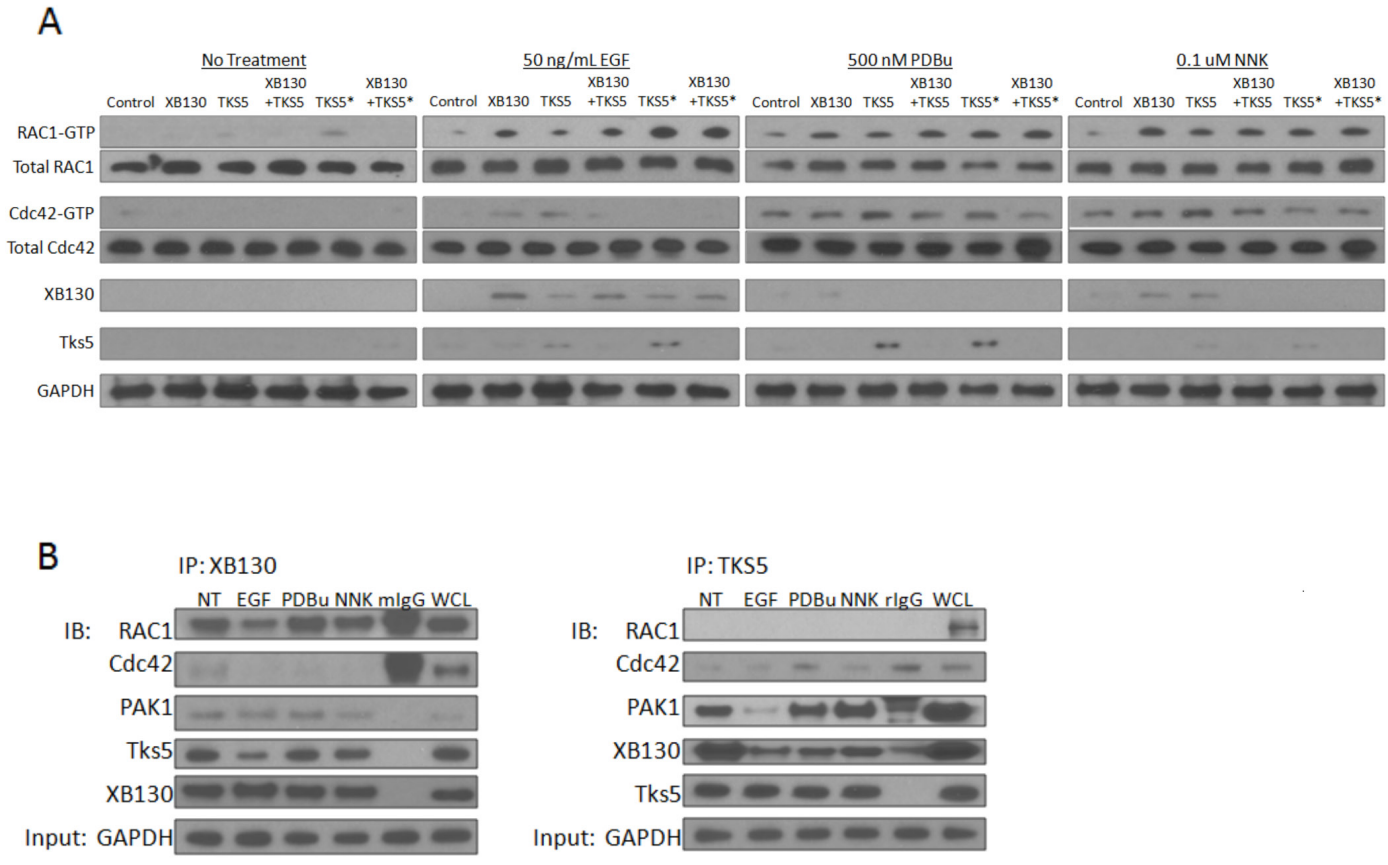
XB130 over-expression enhances Rac1 activation whereas Tks5 over-expression enhances Cdc42 activation **A.** BEAS-2B cells were transfected with GFP/mCherry-vectors (transfection control), GFP-XB130/mCherry vector, GFP/mCherry-Tks5, GFP-XB130 /mCherry-Tks5, GFP/Tks5 SH3#5* or GFP-XB130/Tks5 SH3#5*. Cells were stimulated with or without 50 ng/mL EGF, 500 nM PDBu or 0.1 μM NNK for 1 h. Cell lysates were subjected to GST-PAK-PBD pull-down assay and immunoblotted with Rac1-GTP, Cdc42-GTP, XB130 and Tks5. Total Rac1 and total Cdc42 in cell lysates were blotted for comparison, with GAPDH as a loading control. Rac1-GTP was increased by EGF, PDBu or NNK stimulation, especially in cells over-expressing XB130 alone. Cdc42-GTP increased after PDBu or NNK stimulation and was enhanced by Tks5 over-expression. Rac1-GTP or Cdc42-GTP detection was reduced in cell lysates co-expressing XB130 and Tks5, compared with XB130 or Tks5 alone transfected cells, respectively. Tks5 SH3#5* expression with or without XB130 co-expression rescued RAC-GTP but not Cdc42-GTP expression after stimulation, specifically with EGF. XB130 and Tks5 were only detected in GST-PAK-PBD pull-downs of cells stimulated with EGF, PDBu or NNK, whereas co-expression of XB130 and Tks5 reduced their detection in GST-PAK-PBD pulldowns. **B.** Co-immunoprecipitation of endogenous XB130 or Tks5 and immunoblots of Rac1, Cdc42, PAK1, Tks5 and XB130. XB130 immunoprecipitation effectively pulls down Rac1 under all treatment conditions. By contrast, immunoprecipitation of Tks5 does not pulldown Rac1, weakly pulls down Cdc42 and more effectively pulls down PAK1. Co-immunoprecipitation between XB130 and Tks5 appears to decrease after stimulation with EGF, PDBu or NNK, especially in the Tks5 immunoprecipitation blot. GAPDH is used as a reference to show the initial input of protein concentration used in each immunoprecipitation reaction.

Cytoskeletal remodeling and formation of stimulus-induced cell migration structures are known to be regulated by the Rho GTPases, Rac1 and Cdc42 [28]. We hypothesized that XB130 and Tks5 play distinct functional roles in lung epithelial cell migration by mediating unique Rho GTPase signaling and/or activity. In their GTP-bound active state, Rac1 and Cdc42 form a complex with PAK1 by binding to its N-terminal p21 binding domain (PBD) to promote cell motility [29]. Thus, we performed a GST-PAK-PBD pull-down assay on cells transfected with GFP/mCherry vectors, GFP-XB130/mCherry vector, GFP vector/mCherry-Tks5, GFP-XB130/mCherry-Tks5, GFP vector/Tks5 SH3#5 mutant or GFP-XB130/Tks5 SH3#5 mutant, seeded cells on matrigel and stimulated with 50 ng/mL EGF, 500 nM PDBu or 0.1 μM NNK. Western blot shows that XB130 over-expression enhanced Rac1-GTP expression but did not enhance total Rac1, Cdc42-GTP or total Cdc42 expression, after stimulation with EGF, PDBu or NNK, as compared to control lanes (Figure 7A). In contrast, Tks5 over-expression increased Rac1-GTP and Cdc42-GTP expression but not total Cdc42 nor total Rac1 expression, after PDBu and NNK stimulation, as compared to control lanes (Figure 7A). Tks5 overexpression did not enhance Rac1-GTP expression as compared to XB130 overexpression alone, after EGF stimulation. Co-expression of both XB130 and Tks5 reduced expression of Rac1-GTP, as compared to XB130 overexpression alone, and reduced Cdc42-GTP, as compared to Tks5 overexpression alone, in EGF, PDBu or NNK stimulated cells (Figure 7A). Expression of the mutant Tks5 alone or co-expressed with XB130 increased RAC1-GTP expression, after EGF and PDBu stimulation (Figure 7A).

We then investigated whether XB130 and/or Tks5 interacted with the active PAK/Rho GTPase complexes; PAK/Rac1-GTP or PAK/Cdc42-GTP. Under basal conditions, XB130 and/or Tks5 did not appear to be pulled down by GST-PAK-PBD (Figure 7A). Pull down of GST-PAK-PBD from stimulated cells showed that XB130 was detected only in cells over-expressing XB130 but not in cells co-expressing both XB130 and Tks5 (Figure 7A). Similarly, Tks5 was mainly detected in stimulated cells over-expressing Tks5 alone and to a lesser extent in control or XB130 only expressing cells, but not in cells co-expressing both XB130 and Tks5 (Figure 7A). XB130 co-expressed with Tks5 mutant was unable to rescue XB130 or Tks5 interaction with the active PAK/RhoGTPase complex (Figure 7A).

To investigate the potential binding of endogenous XB130 or Tks5 to Rac1, Cdc42 and PAK, we immunoprecipitated XB130 or Tks5 with cell lysates collected from the no treatment, EGF, PDBu or NNK treated cells grown on matrigel. Immunoblot analysis showed that under all conditions, XB130 interacted with Rac1 but not Cdc42, with a weak interaction with PAK (Figure 7B, left panel). In contrast, Tks5 bound to Cdc42 but not Rac1, and showed an interaction with PAK1 (Figure 7B, right panel). Moreover, Tks5 immunoprecipitation shows that XB130 detection is decreased after stimulation, providing convincing evidence that the endogenous interaction between XB130 and Tks5 decreases after stimulation-induced cell migration (Figure 7B, right panel). XB130 immunoprecipitation also shows a reduction in Tks5 expression in stimulated cells, as compared to non-treated cells (Figure 7B, left panel).

## DISCUSSION

**Figure 8:**
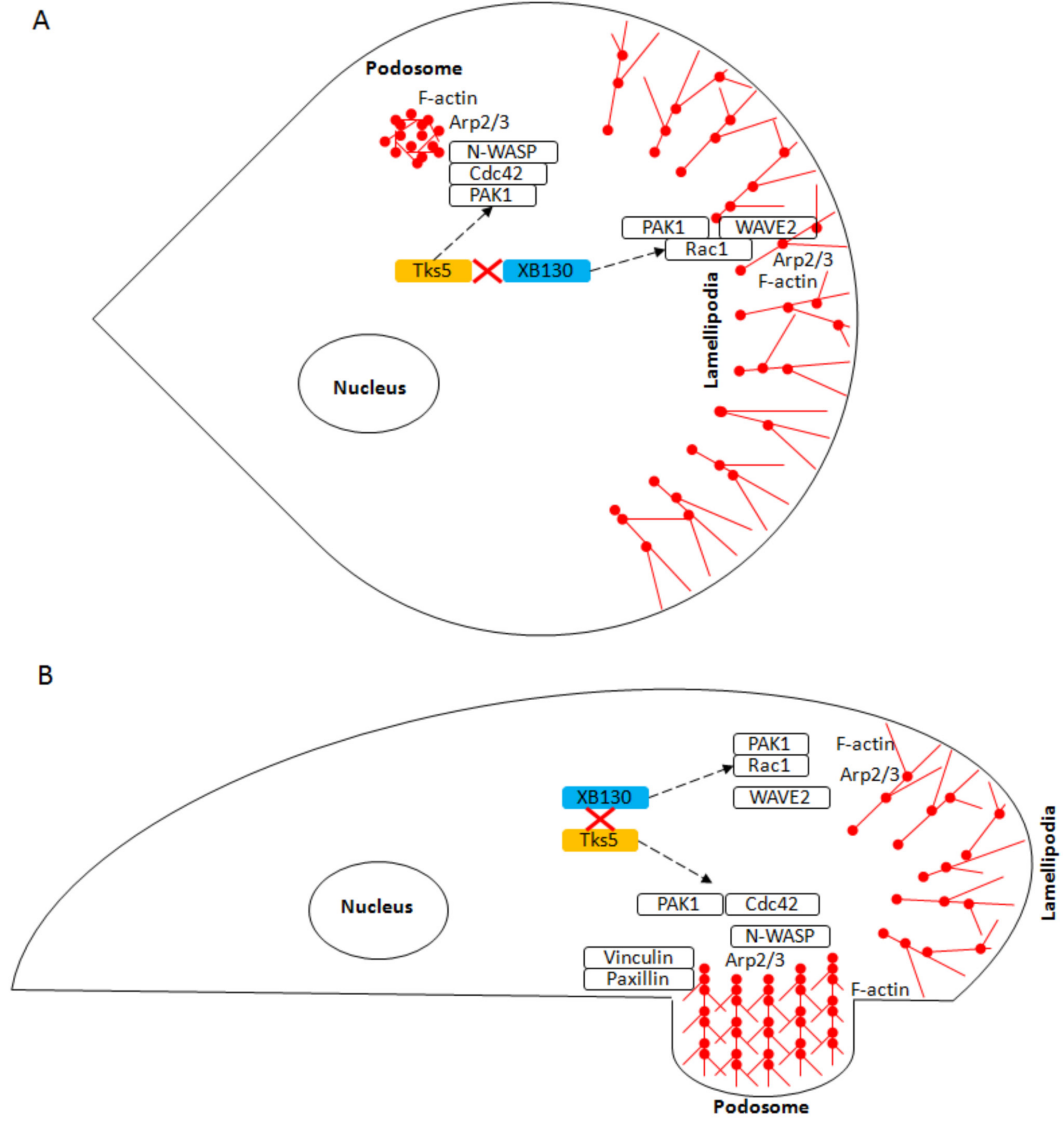
Schematic diagram of the role of XB130 and Tks5 in Rac1 and Cdc42-associated cytoskeletal remodeling for lung epithelial cell migration Cell migration requires cytoskeleton remodeling mediated by the Arp2/3 complex, which results in the formation of branched, F-actin rich structures (red ball and sticks), such as lamellipodia and podosomes. This diagram shows the **A.** Top-down view and **B.** Side-view of a cell displaying lamellipodia and podosome. We demonstrated a novel mechanism for lung epithelial cell migration, in which extracellular factors stimulate a sub-population of XB130 to dissociate from Tks5 and translocate to the cell periphery to promote Rac1-activated signaling of WAVE2-associated lamellipodia formation for cell extension and Tks5 mediation of Cdc42 activity via PAK1 interaction for the promotion of N-WASP-associated podosome assembly and function for ECM-dependent cell migration. Dashed black lines represent translocation of XB130 and Tks5 to the cell membrane.

The scaffold proteins, XB130 and Tks5, are important regulators of various cell functions by interacting with a variety of different proteins for signal transduction [8, 30]. We have shown that XB130 binds via its N-terminal proline-rich motif to the fifth SH3 domain of Tks5, and the interaction between XB130 and Tks5 is important to mediate EGF stimulated cell proliferation and survival in BEAS-2B cells [16]. Herein, we have elucidated a novel stimulus-dependent molecular mechanism for the regulation of lung epithelial cell migration and cytoskeleton remodeling. Stimulation induced XB130/Tks5 dissociation for XB130-mediated Rac1 activation and lamellipodia formation and Tks5-mediated Cdc42 activation, podosome formation and ECM degradation-dependent cell migration (Figure 8). We showed that various factors contribute to distinct XB130- or Tks5-mediated cell migration processes in lung epithelial cells: EGF stimulation drives XB130-mediated lamellipodial formation, whereas, NNK and PDBu contribute to Tks5-mediated ECM degradation-dependent cell migration. The Tks5-mediated increase in ECM degradation-dependent migration is indicative of its role in podosome formation and secretion of proteases for the movement of cells through the substrate. In contrast, XB130 plays a role in promoting lamellipodial formation for adherence and lateral cell migration. XB130 and Tks5 appear to associate endogenously under normal growth conditions for the regulation of cell proliferation and survival, but extracellular perturbation induces dissociation from each other to interact with and activate other proteins for the regulation of cell migration.

Lamellipodia and podosomes are two major cytoskeletal structures that regulate cell migration and invasion, respectively. Many of the same proteins have been found in both structures and a limited subset of proteins is regarded as specific components for either lamellipodia or podosomes. The WASP family is highly integrated into cell migration signaling by binding and activating actin related protein complex (ARP2/3) for nucleation of actin filaments for the formation of actin-rich cytoskeleton structures, like lamellipodia, filopodia and podosomes [31]. Several studies have demonstrated the role of WAVE2 as a marker of lamellipodia that primarily localizes at the leading edge of migrating cells [32]. Similar to observations seen in WAVE2 knockout cells [33], we observed that down regulation of XB130 expression with siRNA effectively inhibits cell migration and the formation of lamellipodia at the leading edge of cells. Immunofluorescence staining shows that both XB130 and WAVE2 translocate to the cell periphery and co-localize robustly at bands along the lamella of cells after stimulation. Stimulation with EGF, PDBu or NNK promotes both XB130 and WAVE2 redistribution from the cytoplasm to cell membrane. As WAVE2 is a marker of lamellipodia, N-WASP is critical for podosome/invadopodia formation [34]. N-WASP has been previously identified as a Tks5 binding partner that regulates actin assembly during podosome assembly [6]. As expected, we observed that both N-WASP and Tks5 localized to podosomes after PDBu or NNK stimulation. In contrast, XB130 did not colocalize with Tks5 or N-WASP at podosomes but rather at the cell periphery. These observations confirm that XB130 is a marker for lamellipodia in cells.

The Rho subfamily of small Ras GTPase proteins is well known for their role as cytoskeleton reorganizers [35]. Two such family members, Rac1 and Cdc42 are both regulators of cell morphology, adhesion and migration. Like WAVE2 and N-WASP, which play contrasting roles, Rac1 is involved in lamellipodial formation, whereas Cdc42 is involved in podosome formation and cell invasion via the N-WASP/Arp2/3 complex [36, 37]. Tks5 expression mediates constitutively active Cdc42 localization within F-actin rich migration structures for assembly of invadosomes and proteolytic degradation of ECM [37]. In cancer cells, XB130 colocalizes with constitutively active Rac1 at lamellipodia [11]. In the present study, we further demonstrated that when normal human airway epithelial cells undergo cytoskeletal reorganization and/or migration, XB130 specifically mediates Rac1 activity but not Cdc42 activity, whereas, Tks5 mainly mediates Cdc42 activity. Moreover, endogenous XB130 binds preferentially to Rac1 over Cdc42, and this explains XB130′s involvement in lamellipodial formation and its role in lateral cell migration, whereas endogenous Tks5 binds to Cdc42 and PAK1, indicating its role in podosome formation and ECM-degradation. XB130 and Tks5 may act in association with GTPase activating proteins to regulate Rac1 and Cdc42 activity, and further study is required to characterize the specific interaction and regulatory function of XB130 and Tks5 with Rac1 and Cdc42.

We previously demonstrated that XB130 binds specifically to Tks5′s fifth SH3 domain endogenously, to regulate cell proliferation and survival in BEAS-2B cells [16]. In this study, the inhibition of enhanced cell migration by co-expression of XB130 and Tks5 and rescue of cell migration by the XB130 binding inhibitor Tks5 SH3#5* suggests that interaction between XB130 and Tks5 may quench Tks5 binding or sequester Tks5 to areas, like lamellipodia with XB130, as we observed in EGF treated cells, thereby preventing Tks5′s interaction with other podosome/ECM degrading-associated proteins. Moreover, enhancement of cell migration by inhibiting the physical binding between XB130 and Tks5 suggests that under specific situations, XB130 and Tks5 dissociate to promote unique cell migration processes. However, the mutant Tks5 did not rescue cell migration to levels observed in Tks5 overexpression nor increase Rac1-GTP and Cdc42-GTP expression and interaction with the PAK/RhoGTPase complex after PDBu and NNK stimulation, suggesting that Tks5 may require the fifth SH3 domain for other protein-protein interactions and cell migration processes. We also observed that XB130 was unable to translocate to podosomes, while Tks5 does co-localize with XB130 at lamellipodia after stimulation, suggesting that XB130 regulates Tks5-mediated cell migration. Additionally, in GST-PAK-PBD pulldowns, co-expression of both XB130 and Tks5 reduces detection of either XB130 or Tks5 in complex with GST-PAK-PBD, further indicating that the XB130/Tks5 interaction prevents interaction of either XB130 or Tks5 with other partners related to cell migration. In contrast, after stimulation, XB130 expression was reduced in the Tks5 immunoprecipitates confirming dissociation of the XB130/Tks5 interaction as a mechanism for the promotion of lung epithelial cell migration.

Most studies on protein-protein interactions predominantly focus on formation of protein complexes. Results from this and our previous studies suggest that temporally and spatially controlled association and dissociation of proteins are critical to regulate complex signaling processes at different stages. We speculate that during normal maintenance of airway epithelia, XB130 and Tks5 interact to regulate cell survival, but upon injury XB130 and Tks5 dissociate to allow each of them to interact with other binding partners to promote cell spreading and migration to rapidly cover the wounded area. Subsequently, the interaction between XB130 and Tks5 is required to promote cell proliferation. Indeed, using XB130 knockout mice, we have found that XB130 plays important roles in tracheal epithelial cell differentiation [12], in promoting bronchial alveolar stem cell proliferation and in lung repair after septic injury [13, 14]. Whether the XB130/Tks5 interaction is involved in XB130-related airway epithelial repair and regeneration should be further studied *in vivo*. Thus, our study provides the foundation to better understand the dynamic protein-protein interactions involved at different stages for the regulation of physiological processes associated with the maintenance and repair of lung epithelium.

## MATERIALS AND METHODS

### Cell culture

Human bronchial epithelial BEAS-2B cells (ATCC, Manassas, VA, USA) were cultured in DMEM low glucose medium (Life Technologies, Rockville, MD) with 10% FBS (GIBCO). All cells were cultured at 37°C with 5% CO_2_ in a humidified cell incubator. For protein expression and immunofluorescence studies, cells were serum starved for 18 h and treated with 50 ng/mL EGF, 0.1 μM NNK or 500 nM PDBu for 1 h at 37°C.

### Reagents, plasmids and antibodies

Small interfering RNA (siRNA) targeting human AFAP1L2, human Tks5 and control siRNA and rabbit anti-N-WASP (H100) (1:750), WAVE2 (H110) (1:500), PAK1 (C-19) (1:250) and mouse anti-Tks5 (M300) (1:500) antibodies were from Santa Cruz Biotechnology (Santa Cruz, CA, USA). XB130 monoclonal antibody (1:2) was generated as previously described [8]. Rabbit anti-XB130 (1:250) and rabbit anti-Tks5 (SH3#1) (1:500) was from EMD Millipore (Merck, Darmstadt, Germany). Anti-GAPDH (1:10000) and Na^+^/K^+^ ATPase (1:20000) antibodies were from Abcam PLC (Cambridge, UK). Rabbit anti-Rac1 (ARC03) (1:250) and mouse anti-Cdc42 (BK034) (1:500) were from Cytoskeleton, Inc. (Denver, CO, USA). Horseradish peroxidase (HRP)-conjugated goat anti-mouse (1:10000) or anti-rabbit secondary (1:10000) antibodies were from Amersham Pharmacia Biotech (Piscataway, NJ, USA). pEGFP-XB130 and p-EGFP-vector were constructed as previously described [11]. mCherry-vector and mCherry-Tks5 were supplied by GeneCopoeia (Rockville, MD, USA). Myc-Tks5 SH3#5 W1108A was provided by S. Courtneidge.

### Cell transfection

Control, XB130 or Tks5 siRNA were transfected into BEAS-2B cells by Oligofectamine (Invitrogen, Carlsbad, CA, USA) following manufacturer's protocol. BEAS-2B cells were seeded in 100 mm cell culture plates. Cells were transfected at approximately 70% confluence and incubated for 48 h before analysis. GFP-vector, GFP-XB130, mCherry-vector and mCherry-Tks5 plasmids were transfected into 80% confluent BEAS-2B cells using Lipofectamine 2000 (Invitrogen) following manufacturer's protocol [16]. Equal concentrations of both GFP- and mCherry-labelled plasmids were transfected into each group of cells and incubated for 48 h before fluorescence activated cell sorting.

### Wound healing assay

Cells were grown to confluence in a 6-well plate and serum starved for 4 h. The cell monolayer was scratched in a straight line and washed to remove debris. Growth media supplemented with 10% FBS was added and cells were incubated at 37°C for 8 h. Images were acquired using phase contrast microscopy at 10x and 40x magnifications at start, 4 h and 8 h. Area and cell number was assessed using ImageJ (National Institutes of Health, Bethesda, MD, USA).

### In situ fluorescent gelatin zymography

Coverslips were coated with 2.5% Oregon green 488 conjugated gelatin (Molecular Probes, Eugene, Oregon, USA). Transfected cells were seeded on the coated cover slips and incubated for 24 h. Cells were serum starved for 4 h and then challenged with 500 nM PDBu over 8 h. Cells were fixed and stained with Texas Red-X phalloidin (Molecular Probes, Eugene Oregon, USA) Images were acquired using Olympus FV1000 confocal laser scanning microscope (Olympus Corporation, Tokyo, Japan) at 60x magnification. Images were quantified using ImageJ (National Institutes of Health).

### Biochemical/immunoblot protein expression studies

Cells were lysed with Triton X-100 lysis buffer or using the FractionPREP Cell Fractionation kit (BioVision Incorporated, Milpitas, CA, USA) for western blot analysis or NP-40 lysis buffer for pulldown procedures. Immunoprecipitation of XB130 and Tks5 was performed as described previously [16]. Protein samples were analyzed using the Pierce 660 nm protein assay (Thermo Scientific, Rockford, IL, USA). SuperSignal Dura Chemiluminescent substrate (Thermo Scientific) was used to detect protein signal after transfer. Contrast and brightness of developed blots were adjusted to display similar background intensity.

### Immunofluorescence microscopy

BEAS-2B cells were seeded on coverslips and incubated overnight in DMEM + 10% FBS at 37°C with 5% CO_2_ in a humidified cell incubator. Cells were fixed using 4% paraformaldehyde and permeabilized with 0.1% Triton X-100 in PBS. Cells were incubated with anti-XB130 (mouse IgG, homemade), anti-Tks5 SH3#1 (rabbit IgG, EMD Millipore), anti-N-WASP (rabbit IgG, Santa Cruz) or anti-WAVE2 (rabbit IgG, Santa Cruz) primary antibody and secondary antibody conjugated with Alexa Fluor 594 (Molecular Probes) or Oregon Green 488 (Molecular Probes). Actin was stained using CytoPainter Phalloidin- iFluor 405 reagent (Abcam, Cambridge, UK). Coverslips were mounted onto glass slides with DAKO fluorescence mounting medium (Dako, Mississauga, Canada). Images were obtained using an Olympus FluoView Confocal, FV1000-ASW and analysed by Olympus FluoView FV10-ASW and ImageJ/FIJI Coloc 2 plugin (National Institutes of Health).

### Transwell migration assays

GFP and mCherry co-transfected cells were collected by trypsinization, centrifuged at 1000xg, resuspended in PBS + 0.1% FBS, passed through a 35 μm meshed cell strainer and sorted using an Aria III CFI (BD Biosciences, San Jose, CA, USA). Cells expressing GFP and mCherry were sorted for enriched double positive staining. Cells were seeded, incubated over 24 h and serum starved for 18 h. Cells were stained with NucBlue Live Ready Probes reagent (Thermo Fisher Scientific, Waltham, MA, USA) and seeded in serum free media into 24-well 8.0 μm transwell permeable supports (Corning Costar, Corning, NY, USA). Transwells were coated with 200 μg/mL phenol-red free, growth factor reduced matrigel (Corning Costar). Cells were treated with 50 ng/mL EGF, 0.1 μM NNK or 500 nM PDBu at 37°C for 1 h. Media in the 24-well plate was replaced with normal growth media and cells were incubated at 37°C for 8 h for uncoated transwells or 24 h for matrigel coated transwells. Permeable transwell supports were washed and placed in serum-free media. Live cell, fluorescent images were acquired using a Nikon A1R single photon confocal microscope (Nikon Corporation, Tokyo, Japan). Images were analysed using NIS Elements (Nikon Corporation, Tokyo, Japan), ImageJ (National Institutes of Health) and Imaris (Bitplane AG, Zurich, Switzerland).

### Rac1/Cdc42 activity assay

Previously sorted GFP and mCherry positive cells were seeded, stimulated and recovered from matrigel. Recovery from matrigel utilized ice cold PBS and gentle mechanical separation by pipette. Cells were centrifuged at 1000xg for 1 min and washed. Cdc42 Activation Assay Biochem Kit (Cytoskeleton, Inc., Denver, CO, USA) was used to process cells. For each pulldown sample, 0.5 mg/mL total cell lysate was incubated with 10 ug PAK-PBD beads and the assay protocol was followed according to kit instructions. Samples were run on a 4-12% Bolt Bis-Tris Plus SDS-PAGE gel (Novex, Life Technologies, Thermo Fisher Scientific, Waltham, MA, USA), transferred to PVDF membrane and blotted for Cdc42, Rac1, XB130 and Tks5.

### Statistical analysis

Data are expressed as mean ± SD and analyzed by two-way analysis of variance or student's t-test with α ≤ 0.05 using GraphPad Prism 5 (GraphPad Software Inc., CA, USA). Bonferroni correction was used to establish a significance of *p* ≤ 0.01. Using Image J Coloc2 Plugin, regions of interest were selected and mapped and the Manders split co-efficient with Costes significance testing was selected to analyze the amount of fluorescence of the colocalizing pixels in each colour channel. Values range from 0 to 1 and express the amount of signal intensity shared by both colour channels in a given pixel area.
